# Cellulose–xylan composite fibres as precursors for carbon fibres

**DOI:** 10.1039/d5ra07682j

**Published:** 2025-12-08

**Authors:** Mikaela Trogen, Petri Kilpeläinen, Leena Pitkänen, Daisuke Sawada, Lukas Fliri, Inge Schlapp-Hackl, Michael Hummel

**Affiliations:** a Aalto University, School of Chemical Engineering, Department of Bioproducts and Biosystems P.O. Box 16300 00076 Aalto Finland michael.hummel@aalto.fi; b Natural Resources Institute Finland (Luke) Latokartanonkaari 9 00790 Helsinki Finland; c Department of Chemistry, Institute of Chemistry of Renewable Resources, BOKU University Konrad-Lorenz-Strasse 24 3430 Tulln Austria

## Abstract

Carbon fibre reinforced composites are used to provide high-strength light-weight materials that are sought after in mobility applications to reduce weight and consequently fuel or energy consumption of the transportation vehicle. Cost and environmental considerations have resurged research on biobased precursors for carbon fibres (CFs) with cellulose being one of the most prominent examples. In this study we shed light on the purity requirements of the cellulose substrate. Thus far, most reports on lignocellulose-based CFs implement highly refined dissolving-grade pulp. To reduce the cost of the precursor fibres and their environmental footprint even further, less refined cellulose sources are desirable. The role of xylan on the properties of the precursor fibres and the carbonization behaviour of holocellulosic precursor fibres were studied. It was found that natively present hemicelluloses in paper-grade kraft pulp can be incorporated homogeneously into the cellulose matrix without impairing the fibre properties, and even showing a beneficial effect on the final carbon yield.

## Abstract 2

Native hemicellulose can increase the carbon yield of biobased carbon fibers if hemicellulose is retained and evenly distributed in the precursor fibers.

## Graphical abstract

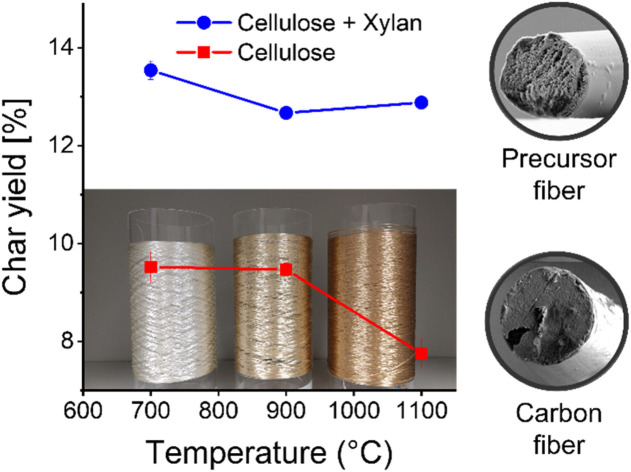

## 1 Introduction

Concerns regarding environmental issues and climate change have shifted the focus towards utilising more sustainable resources for production of various goods. They have also enforced a need to decrease both fossil fuel consumption and emissions. To fulfil these goals, we need lighter, energy-efficient vehicles, as well as raw materials and fuels from renewable resources. For both, electric and fossil driven vehicles the range as well as the energy consumption depend on the vehicle weight. Choi, Cha and Song reported that a 15% increase in the weight of the electric vehicle resulted in a 4–9% increase in wheel energy consumption.^1^ Carbon fibres (CFs), with a desirable weight-to-strength ratio, play an important role in producing light-weight vehicles, with reduced energy consumption. In electric vehicles, the battery pack constitutes a significant portion of its weight, often accounting for 20–25% of its curb weight.^1^ Therefore, not only the chassis of the vehicle should be considered, but also novel battery solutions need to be developed. Structural batteries, with multiple functions, serving both as a load-bearing structural component and as an energy storage device could greatly reduce the weight of electric vehicles.^2^

Cellulose fibres were already considered as precursors for carbon fibre (CF) production in the 1950s. The discovery of polyacrylonitrile (PAN) as a CF precursor, quickly displaced cellulose and very soon PAN became the preferred precursor, due to the superior properties of PAN-based CFs. There are, however, some challenges in the use of PAN-based CFs. They come with excellent properties, but accompanied with a high price, and the manufacturing process also involves emission of toxic gases (for example HCN). An extensive overview of CF production and precursors has been published by Frank *et al.*^3^ The excellent and broadly adjustable mechanical properties of PAN-based CFs are needed for high-end applications and justifies their relatively high production costs. However, it limits high-volume low-price applications, such as parts for the car industry, in which CFs with medium strength properties would suffice. Cheaper CFs would allow production of more light-weight car body parts to reduce fuel or electricity consumption. Since PAN is produced from non-renewable materials, the focus is again shifting towards finding CF precursors from renewable resources.

In recent years, a growing number of research projects have focused on producing CFs from lignocellulosic precursors. Several studies on cellulose precursor fibres (PFs)^4,5^ and cellulose–lignin composite fibres as precursors for CFs^6–13^ have been published recently, but work focusing on hemicelluloses^14–17^ in the precursor fibres is quite scarce. To produce continuous CFs, continuous PFs are needed. The first available manufactured cellulose filaments were produced *via* the viscose process. Classical viscose fibres, however, had intrinsic properties that were not beneficial for the production of high-quality carbon fibres. An uneven cross section impeded homogeneous carbonization, and a low molecular orientation of the cellulose molecules in the fibre matrix limited the formation of a highly ordered carbon structure. Lyocell-type cellulose fibres developed from the 1970s onwards and commercialized in the 1990s could overcome those limitations. Recently, Lenzing AG presented a patent for producing Lyocell continuous fibres at high speeds, up to 700 m min^−1^ or higher.^18^ The main end use for these filaments would be in textile applications, but this process could also be suitable for producing PFs for CF production.

In the last ten years, a new Lyocell-type spinning process termed Ioncell® has been developed.^19,20^ The biopolymers are dissolved in a super-base based protic ionic liquid (IL), (*e.g.*, 1,5-diazabicyclo[4.3.0]non-5-ene acetate, ([DBNH] OAc) and the fibres are dry-jet wet spun. Essentially all wood biopolymers can be dissolved in this IL. Fibres containing cellulose,^21^ hemicellulose,^15^ and lignin^9,13,22^ have been spun successfully. The main target of the Ioncell® research has been set on textile fibre production.^23,24^ Nevertheless, Ioncell® fibres are also suitable to produce carbon fibres.^4,8–11^

Pure cellulose PFs give a low CF yield, with the theoretical maximum of only 44.4%.^3^ Lignin possesses a significantly higher carbon content (60–65%)^25,26^ than cellulose, so the CF yield can be increased by adding lignin to the PFs.^9,12^ The carbon content of xylan varies with the structure of xylan in different wood species. Sharma *et al.* reported a carbon content of 36.70% for extracted acacia xylan^27^ and Liu *et al.* reported 41.40% of carbon in beech xylan.^28^ Comparing the theoretical maximum carbon yields of cellulose and xylan in CFs, the carbon yield of xylan appears slightly lower. However, a notable loss of carbon takes place through levoglucosan formation. The primary C6-OH-group in the cellulose backbone attacks the anomeric carbon and cleaves the glycosidic bond.^29,30^ The loss of levoglucosan in the pyrolysis process renders the actual char yield of cellulose much lower than the theoretical yield. Bengtsson *et al.* reported yields for CFs produced from softwood (SW) paper-grade pulp and SW dissolving pulp of 21% and 22% respectively.^12^ Byrne *et al.* reported a char yield of 26% in thermogravimetric analysis of cotton fibres.^31^ Morais de Carvalho *et al.* reported a char residue of 32.8 ± 0.6% for alkali-soluble birch xylan and 31.5 ± 0.1% for a commercial birch xylan with thermogravimetric analysis at 600 °C.^32^ Hardwood xylan lacks the C6-OH group, thus levoglucosan formation is not possible. Due to the structural difference of xylan and cellulose, pyrolysis of xylan will result in furan derivatives instead of levoglucosan.^30^ Zhu *et al.* reported five primary pyrolytic products. Depolymerisation of xylan resulted in furfural, ring scission produced hydroxyacetaldehyde, 1-hydroxy-2-propanone and 1-hydroxy-2-butanone, and cleavage of acetyl groups generated acetic acid.^33^ Since the formation of levoglucosan is inhibited, adding xylan to PFs, could lead to a higher overall CF yield.

Herein we present a systematic study on xylan-containing cellulosic PFs for CF production. The work includes characterisation of raw materials as well as produced PFs and CFs. This work is a continuation to our previously published work on cellulose–lignin fibres.^9,10^

## 2 Experimental

### 2.1 Materials

Pre-hydrolysed kraft birch pulp (E), ([*η*] = 494 ml g^−1^), from Stora Enso Enocell mill in Finland and birch xylan from Natural Resources Institute Finland (Luke) were used for the fibre preparation. The xylan was utilised as received. The preparation of the xylan samples is described in Section 2.2. In addition, also a hemicellulose-containing paper-grade (PG) birch pulp ([*η*] = 800 ml g^−1^) from UPM Kaukas mill in Finland was used to prepare PFs. The pulp sheets were ground to a fine powder, using a Wiley mill. 1,5-diazabicyclo[4.3.0]non-5-ene (DBN) (Fluorochem, UK) and acetic acid (glacial, 100%, Merck, Germany) were used to prepare the IL. DBN and acetic acid were used as received. The spin bath was filled with tap water (9 ± 2 °C).

### 2.2 Xylan preparation

The sawdust was collected from a sawmill and stored at −20 °C in a freezer before extractions. Birch sawdust was extracted with a pressurized hot water extraction system.^34^ The sawdust sample (134.1 kg fresh, 62.9 kg o.d.) was added into the reactor. Pressurized hot-water flow-through extraction was conducted at 170 °C for 60 minutes with 20 l min^−1^ flow. Totally 1037.5 kg of extract was collected. The extract was concentrated and purified with ultrafiltration.^35^ 979.5 kg of extract was ultrafiltered with polyethylesulphone membranes (EM006, PCI membranes) until 52.4 kg of concentrated extract was obtained. Three different birch glucuronoxylan samples were prepared from the concentrate with different monosaccharide and lignin content.

#### 2.2.1 Technical xylan

The first batch (Xylan Technical, XylTech) was prepared from concentrated extract (21.21% d.w.) that was spray dried with a GEA Niro A/S Mobile Minor 0.8 2008 spray drier. The concentrate (5.9 kg) was dried at 200 °C (inlet temperature) and the feed flow was adjusted so that the final temperature of the outlet was 70 °C. A total of 0.97 kg of dry product (XylTech) was collected.

#### 2.2.2 Precipitated xylan

The second glucuronoxylan sample (Xylan Precipitated, XylPrec) was prepared by ethanol precipitation. The concentrated extract was added slowly to the vigorously stirred ethanol phase (Etax Aa, Altia Finland). The ratio of extract and ethanol were 1/10 vol/vol. Precipitated xylan with 15% dry weight was purified using pressure filtration (Ertelalshop LAB10T). One litre of precipitate and 200 ml of ethanol were added into the system in each run. The precipitate was washed with 550 ml of ethanol three times, until a clear filtrate was obtained. The purified precipitate was dried in a vacuum oven overnight at 40 °C. The process was repeated four times to obtain enough XylPrec sample for further experiments.

#### 2.2.3 Purified xylan

The third sample (Xylan Purified, XylPur) was prepared by further purifying 47.0 g of ethanol precipitated glucuronoxylan sample (XylPrec) with 500 ml of 20–60 mesh XAD7HP adsorbent (Sigma-Aldrich, France) loaded into a column. The column was washed with 400 ml of deionized water before the experiment. The sample was dissolved in 470 ml of deionized water and fed to the column. Effluent was collected 6.5 min after the liquid sample was fed into the column. After the sample was fed to the column, deionized water was added, to elute compounds from the column. In total, 1174.5 g of effluent were collected with 30.05 g (64% of fed sample) of original material and was further concentrated on a heating plate under nitrogen flow. Concentrated liquid was precipitated with ethanol (1/10, vol/vol) and dried overnight in a vacuum oven at 40 °C to produce the purified sample (XylPur).

### 2.3 Spinning process

The spinning process was performed according to procedures described in detail in our previous work.^9^ The only modification was in the preparation of IL. The IL 1,5-diazabicyclo[4.3.0]non-5-enium acetate ([DBNH]OAc) was prepared in a 6 L-glass reactor with heating (70 °C) and after the slow addition of acid the solution was mixed for 30 min. The IL was stored in glass bottles at room temperature. Before use, the bottles were kept in a water bath at 90 °C for 90 min, allowing the IL to melt. Pulp and xylan were dissolved in the IL using a vertical kneader (80 °C, 90 min (120 min for PG), 110 ± 20 mbar, mixing 30 rpm). The cellulose–xylan dopes were filtered (filter pore size 10 µm) using a filter press. The filtered dope was spun using a piston-spinning unit (Fourné Polymertechnik, Germany), with an air gap of 1 cm (dry-jet wet spinning). A spinneret with 400 holes, capillary diameter 100 µm and L/D 0.02 was used. The extrusion rate in the spinning process was 5.5 ml min^−1^ (11.0 ml min^−1^ for PG) and the temperature 65 ± 5 °C (96 ± 2 °C for PG). PFs with different cellulose–xylan ratios and different draw ratios (DR) were spun (Table 1). After spinning, the fibres were washed with tap water using a custom-made washing line (washing: 10 ± 0.25 min retention time at 68 ± 3 °C, drying: 80 °C). Different total solids concentrations of the dopes and different spinning temperatures were used, in order to achieve rheological properties suitable for spinning.

**Table 1:** Precursor fibres

PG pulp [%]	E pulp [%]	Xylan [%]	Xylan type	Dope total conc. [%]	Fibre code	DR^a^
—	100	0	—	13	E100	3, 6 and 12
—	90	10	Technical	13	E90-XylTech10	3, 6 and 12
—	70	30	Technical	15	E70-XylTech30	3, 6 and 12
—	90	10	Precipitated	13	E90-XylPrec10	3, 6 and 12
—	70	30	Precipitated	15	E70-XylPrec30	3, 6 and 12
—	90	10	Purified	13	E90-XylPur10	3, 6 and 12
100	—	—	—	10	PG100	3, 6 and 12

a
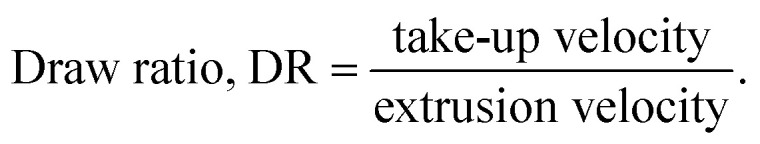

### 2.4 Carbonization

The carbonization tests were performed using a tube furnace (Nabertherm RHTH 80-300/16, Nabertherm GmbH, Germany) under nitrogen gas flow (100 l h^−1^). Six different temperature programmes were used (Table 2). The PFs were carbonized without any pre-treatment, and no tension was applied on the fibres during carbonization.

**Table 2:** Carbonization programmes

Prog.	Starting temperature [°C]	1st Heating rate [°C min^−1^]	Stabilisation temperature [°C]	Hold time [min]	2nd Heating rate [°C min^−1^]	Final temperature [°C]	Hold time [min]
1	0	5	—	—	—	700	60
2	0	5	—	—	—	900	60
3	0	5	—	—	—	1100	60
4	0	2	250	30	—	—	—
5	0	2	250	30	5	700	30
6	0	2	250	30	5	1100	30

### 2.5 Analyses

Carbohydrate–lignin content and molar mass distribution of raw materials and fibres, as well as the mechanical properties and birefringence of the fibres were determined according to the procedures described in our recently published work.^9^ SEM images were also taken according to recently published procedures.^9^ Detailed description of the procedures can be found in the SI.

The elemental composition (Elemental Analysis, EA) of all investigated materials was analysed by combustion EA using a Thermo Flash Smart CHNSO Elemental Analyzer. Starting materials and fibres which did not undergo heat treatment where dried at 105 °C overnight to limit water contaminations. Measurements for thermally stabilized PFs and CFs were performed under addition of 8–10 mg V_2_O_5_ as combustion aid, to assure full conversion. The device was calibrated by a linear calibration using 2,5-bis(5-*tert*-butyl-2-benzo-oxazol-2-yl) thiophene (BBOT) as standard, conducted separately for all performed runs. All measurements were taken at least in triplicate and averaged. Elements C, H, N and S were analysed directly. Results for elements C, H and N are presented here.

WAXS experiments were performed in transmission mode using a Xenocs Xeuss 3.0 CuKα X-ray instrument operated at 50 kV and 0.6 mA. Fibre samples were placed vertically on the sample holder and analysed under vacuum conditions (=0.16 mbar). The scattering profiles were recorded on a 2D Dectris Eiger2 R 1M detector with a sample-to-detector distance of 56 mm, and the scattering intensities were corrected for the cosmic background with a blank run. The 2D scattering patterns were processed using pyFAI software.^36^ For cellulose fibre samples, the 2D scattering patterns were analysed to obtain structural parameters of the cellulose crystal as previously described.^9^ Herman's orientation parameter, crystallinity index, crystal sizes, and the *d*-spacing were estimated. For the carbon fibre samples, the intensity profile (*I*(*Q*) *vs. Q*) was used to estimate the crystallite size *L*_*a*_ and *L*_*c*_ (shape factors of *K* = 0.9 and *K* = 1.84) by the Scherrer equation according to Lundahl *et al.*^37^ The *d*-spacing was calculated by the Bragg equation, and the crystallinity index by the estimation of the ratio of the area of total intensity and background intensity over a range from 9 to 50° 2*θ*.^38^

Thermogravimetric analysis (TGA) was performed using a Netzsch STA 449 F3 Jupiter & QMS 403 Aëolos Quadro thermal analyser with helium atmosphere (70 ml min^−1^). The heating rate was 10 °C min^−1^ and the temperature ramp was set from 40 °C to 800 °C.

Raman spectroscopy was carried out using a Renishaw inVia™ confocal Raman microscope, using a green laser at 532 nm and a 20× objective lens, with 0.3 s exposure time. The measurements were carried out at a spectrum range of 790–1935 cm^−1^. Each sample was analysed in triplicate.

## 3 Results and discussion

### 3.1 Raw materials and precursor fibres

Raw materials, as well as produced PFs and CFs were analysed in order to investigate the influence of the raw materials on the properties of the produced PFs and CFs. Elemental analysis of raw materials and PFs was performed, and the results are summarised in Table 3. The carbon content of all samples varies from 41.7 to 42.7%. For cellulose samples, this agrees fairly well with the theoretical carbon content of 44.4%.^3^ A carbon content of 42.3% for birch xylan also falls in the same range with reported carbon content of beech xylan.^28^

**Table 3:** Elemental analysis of raw materials and PFs

Sample	N [%]	±	C [%]	±	H [%]	±
Enocell pulp	0.0	0.0	42.6	0.1	6.2	0.1
PG pulp	0.0	0.0	42.7	0.2	6.2	0.1
Xylan purified	0.0	0.0	42.3	0.3	6.0	0.1
E100 DR6	0.0	0.0	41.9	0.1	6.3	0.1
PG100 DR6	0.0	0.0	41.7	0.1	6.1	0.1

The carbohydrate and lignin contents of the raw materials are presented in Table 4. The Enocell dissolving pulp (E) contains only minor amounts of lignin and 7.6% hemicelluloses, while the paper-grade pulp (PG) contains 26.3% hemicelluloses and 1.2% lignin. These results were in line with earlier published data for dissolving pulp^39^ and paper-grade pulp.^40,41^ The hemicellulose content of the xylan samples varies with the level of purification, from 75.2% in the technical sample to 92.0% in the purified sample.

**Table 4:** Carbohydrate–lignin content of raw materials

Raw material	Cellulose [%]	Hemicelluloses [%]	Lignin [%]
Enocell pulp	91.8	7.6	0.7
Xylan technical	1.1	75.2	23.7
Xylan precipitated	3.3	88.2	8.4
Xylan purified	4.5	92.0	3.5
PG pulp	72.6	26.3	1.2

**Fig. 1:**
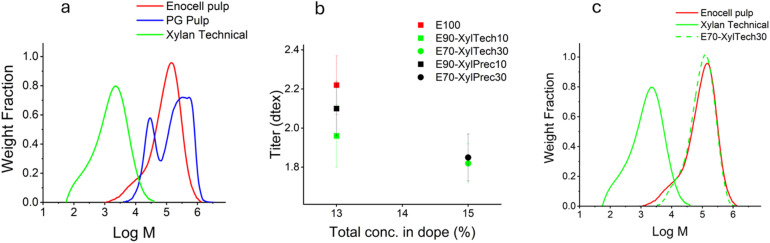
Molar mass distribution and titre of selected samples. (a) Molar mass distribution of raw materials. (b) Titre of fibres spun at DR 6 from solutions with different polymer concentrations. (c) Molar mass distribution of Enocell pulp, the technical xylan, and fibres spun from a mixture of both.

Fig. 1a shows the molar mass distribution of the dissolving and paper grade pulps and the technical xylan. The xylan sample exhibits significantly lower molar mass compared to the dissolving pulp (Enocell). The value agrees well with previously published data.^42^ Typical for paper-grade pulp, it shows two separate peaks, the lower molar-mass peak for the hemicelluloses portion and the higher molar-mass peak representing the cellulose part. The curve for the paper-grade pulp corresponds well to the data published by Borrega *et al.*^43^ However, the molar mass for the cellulose part in the PG pulp is higher than shown in Fig. 1a. The limit of the gel permeation chromatography column was reached at 1000 kDa and all larger molecules eluted at the same time. The unrefined hemicellulose present in the PG pulp has a significantly higher molar mass than the isolated technical xylan.

The carbohydrate and lignin content of the spun fibres were also determined. The measured and the theoretical values are shown in Table 5. The theoretical values were calculated using the values for the raw materials and the portion of pulp and added xylan used in the PF. The fibres with added xylan and the fibres spun from pure Enocell pulp showed almost identical compositions, indicating that the added xylan did not coagulate alongside the cellulose in the fibre.

**Table 5:** Carbohydrate–lignin content of precursor fibres

Sample	Measured			Theoretical		
	Cellulose [%]	Hemicelluloses [%]	Lignin [%]	Cellulose [%]	Hemicelluloses [%]	Lignin [%]
E100	91.9	7.5	0.6	91.8	7.6	0.7
E90-XylTech10	89.4	7.1	3.5	82.7	14.3	3.0
E70-XylTech30	88.7	7.8	3.6	64.6	27.8	7.6
E90-XylPrec10	89.3	7.0	3.7	82.9	15.6	1.5
E70-XylPrec30	89.1	7.9	2.9	65.2	31.8	3.0
E90-XylPur10	90.1	7.1	2.8	83.0	16.0	1.0
PG100	74.1	24.9	1.0	72.6	26.3	1.2

The fibre diameter of the fibres with added xylan was smaller than expected. As the total concentration of the dope increases, the titre of the fibres should increase. However, as shown in Fig. 1b, the titre decreased with increasing xylan content and total polymer concentration. This also supports the observation that a significant portion of xylan was lost during the spinning process. The xylan did not coagulate with the cellulose, instead it remained dissolved in the spin bath.

Nypelö *et al.*^15^ also reported smaller diameters of fibres containing xylan, compared to cellulose fibres spun from dopes with the same polymer concentration. Although, they did not determine the composition of both the raw materials and of the fibres, the fibres showed a lower hemicellulose content than would be the theoretical value. Therefore, it is likely that the smaller diameter of the fibres originated from loss of xylan in the spinning process.

The hemicellulose content of the fibres spun from PG pulp was only slightly lower (24.9%) compared to the original PG pulp (26.3%) (Table 5). The molar mass of the technical xylan was significantly smaller than the molar mass of the xylan found in the PG pulp (Fig. 1a), which likely explains why the added xylan remained dissolved, while the xylan in the PG pulp regenerated with the cellulose. It was shown earlier that the solubility of carbohydrates in ionic liquid-water mixtures depends on the molecular weight, with cellulose and hemicelluloses of low molar mass being more soluble.^44–46^ Hemicelluloses that remain after the pulping process dissolve and coagulate with the cellulose in the Ioncell® process. This is in agreement with findings of Bengtsson *et al.* for fibres from SW paper-grade pulp.^12^ Dammström *et al.* found that some of the xylan is easily extracted, while another part stays intact. They also speculated that the xylan fraction that remained intact was strongly associated with cellulose and that the xylan lost in the pulping process was associated with lignin.^47^ This could explain why the xylan left in the PG pulp dissolved and regenerated with the cellulose throughout the spinning process, while the added xylans failed to do so.

The molar mass distribution of the spun fibre (E70-XylTech30) is very similar to the molar mass distribution of the Enocell pulp (Fig. 1c). This also confirms that xylan did not regenerate with the cellulose. Only a subtle shoulder is visible on the lower molar mass side of the molar mass distribution curve. These are the hemicelluloses that were present in the Enocell pulp, the added technical xylan was virtually lost entirely.

**Fig. 2:**
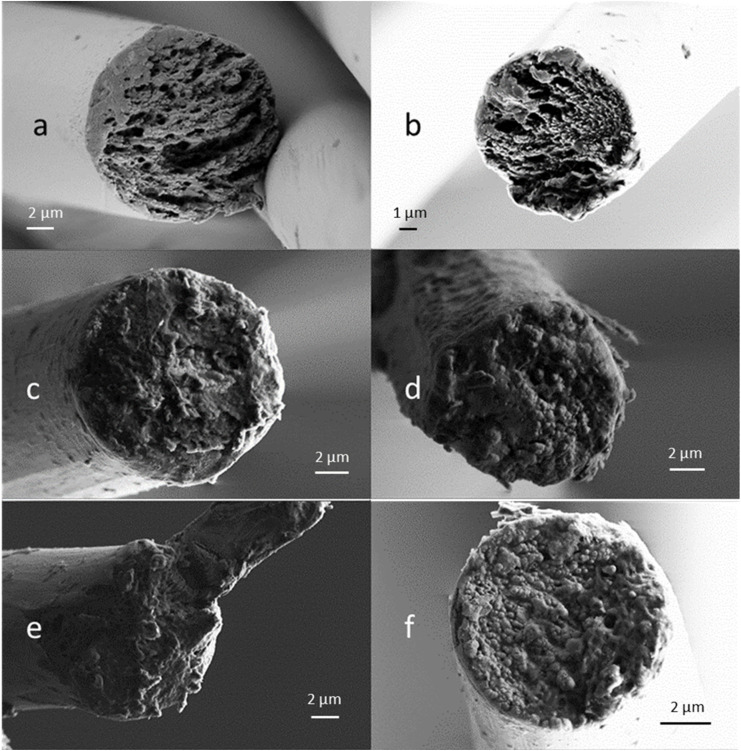
SEM images of selected PFs. (a) Enocell DR6, (b) Enocell DR12, (c) E70-XylPrec30 DR6, (d) E70-XylPrec30 DR12, (e) E70-XylTech30 DR6, (f) 70-XylTech30 DR12.

The crystalline parameters and the total orientation of the fibres (Table 6) show no significant differences between the sample E100 and the samples with added xylan. A small decrease of the C.I.EQ values for the samples E70-XylTech30 and E70-XylPrec30 was observed. This might be a result of the hemicelluloses not co-coagulating with the cellulose fraction during the solidification process, but it could also originate from the slightly higher lignin content of these fibres (Table 5).^9^ It appears that xylan leaves the polymer matrix instantly with the ionic liquid and does not affect the coagulation process of the cellulose fraction on a macromolecular level. However, there were some subtle changes in the microfibrillar structure of the resulting fibres. SEM images of pure cellulose fibres showed the typical round cross section and fibrillary fibre body structure (Fig. 2).^21^ The fibres resulting from a mixed cellulose–hemicellulose substrate also had a round cross section typical of Lyocell-type fibres. However, the fibrillar structure, although clearly visible, was less distinct and contained spherulite-like features. Possibly, these are a result of a reduced diffusion rate of the ionic liquid during the coagulation process. Since hemicellulose remain soluble in the IL-water mixture, the viscosity is slightly elevated which would slow down the diffusion of the IL from the nascent solid fibre into the coagulation bath.

**Table 6:** Crystallinity parameters of PFs^a^^,^^b^^,^^c^

Fibre	DR	*f*_WAXD_	C. I. EQ	Ave. CW [Å]	Total orientation
E100	6	0.84	55	37	0.75
PG100	6	0.81	51	37	0.75
E70-XylTech30	6	0.80	49	38	0.71
E70-XylPrec30	6	0.82	51	37	0.70

a C. I. EQ.: Crystallinity index from equatorial scattering of fibre diffraction.

b *f*_WAXD_: Hermans orientation parameter.

c Ave. CW: Average width of three crystal lattice planes.

**Fig. 3:**
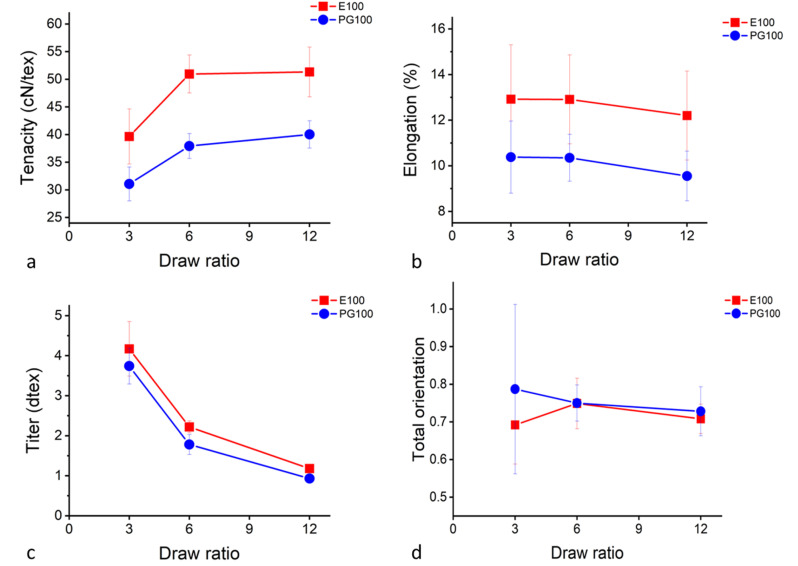
Fibre properties of PFs. (a) Tenacity, (b) elongation, (c) titre, and (d) total orientation as a function of the draw ratio.

Since the addition of xylan did not result in mixed cellulose–hemicellulose fibres, further studies focused on the xylan-containing PFs produced from paper-grade pulp (PG100) and PFs produced from Enocell dissolving pulp (E100) as reference. The mechanical properties as a function of the draw ratio are illustrated in Fig. 3a–c. Already at DR 6, the fibre properties had developed and the tenacity for the Enocell fibres reached a value of approximately 50 cN/tex. This agrees with earlier published values. Nypelö *et al.* also presented values around 50 cN/tex for Enocell fibres^15^ and Ma *et al.* reported a tenacity of 50.5 ± 4.8 at DR 17.7 for fibres made from Eucalyptus dissolving pulp.^22^ The birefringence of the E100 and the PG100 samples was measured, and the total orientation calculated. Both E100 and PG100 fibres showed a total orientation between 0.70 and 0.75 (Fig. 3d). Measuring the total orientation of the fibres with DR 3 was difficult, resulting in high standard deviations. Ma *et al.* reported a total orientation of 0.730 for fibres (DR 12.4) made from Eucalyptus pre-hydrolysed kraft pulp.^22^

**Fig. 4:**
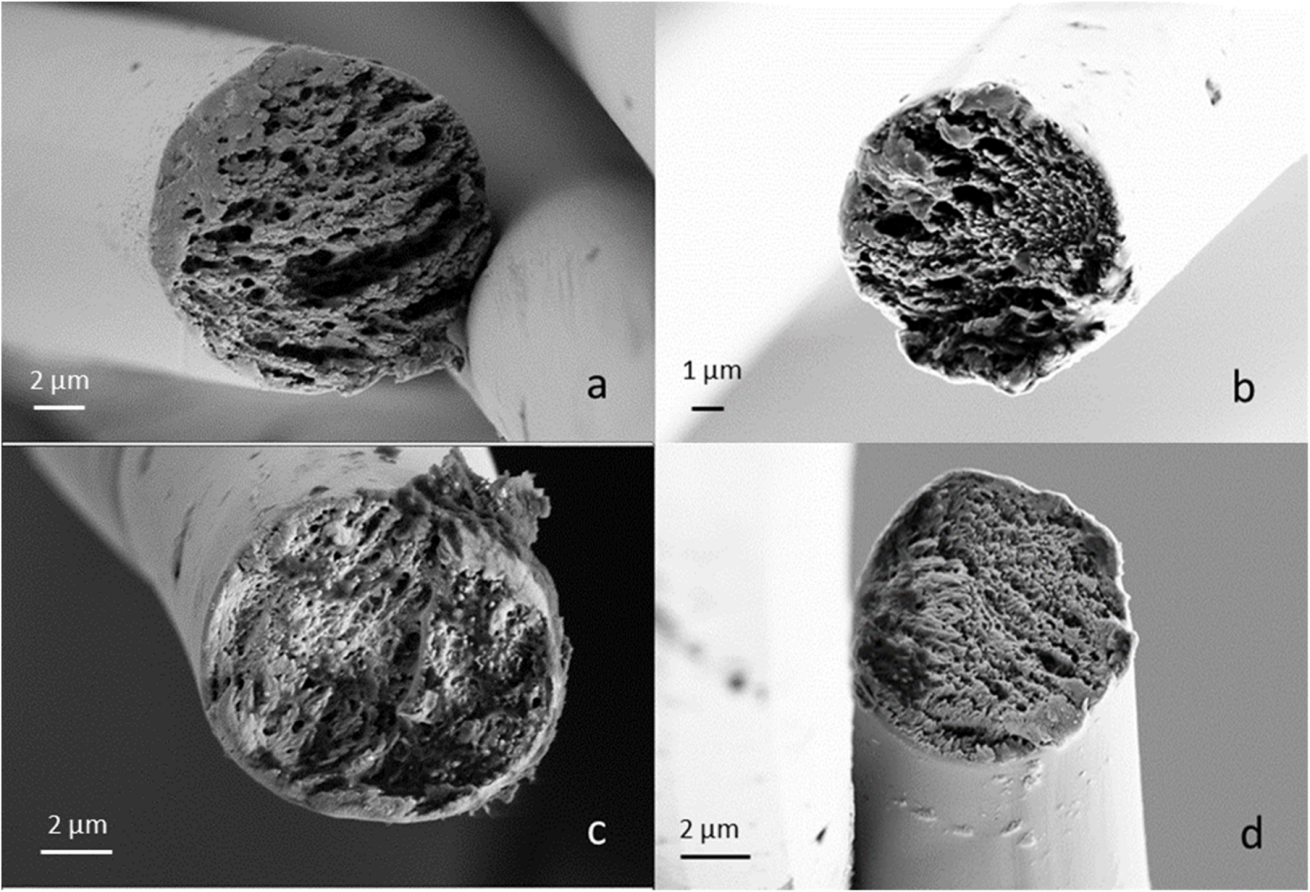
SEM images of selected PFs. (a) E100 DR6, (b) E100 DR12, (c) PG100 DR6 and (d) PG100 DR12.

The high orientation can also be seen in the SEM images. All fibres show a fibrillar cross section both at DR 6 and DR 12 (Fig. 4), supporting the findings that the fibre properties develop already at lower DRs than DR 6 (Fig. 3a). Also, no spherulite structures were visible like in the case of 70-XylTech30 (Fig. 2).

### 3.2 Carbonization and carbon fibres

**Fig. 5:**
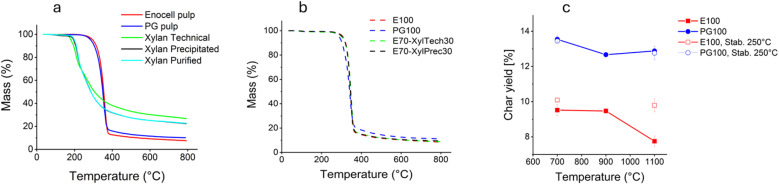
TGA curves of (a) raw materials (b) selected PFs and (c) char yield of fibres after carbonization in a tube furnace.

Prior to carbonization in stationary furnaces, the pyrolysis behaviour of the starting materials and different PF samples was studied *via* TGA (Fig. 5a and b). The residual mass at 800 °C was higher in the case of the xylan samples compared to the pulp samples (Fig. 5a). This is in line with earlier observations by Patwardhan *et al.*^48^ Due to the lack of the C-6 hydroxy group the formation of levoglucosan is not possible, preventing one major source of carbon loss. Liu *et al.* reported similar TGA curves for xylan isolated from corn stalks.^49^ However, the TGA curves for the xylan samples must be interpreted with caution, since we observed that the xylan samples ballooned during the analysis, forming a charred surface that acted as an insulator. Thus, the pyrolysis might have been incomplete in some parts of the bulk phases. This phenomenon was observed for all xylan samples. Ballooning also occurred when trying to measure the ash content of the samples in a box furnace under air atmosphere. Fig. 5b depicts the TGA curves of selected PFs. Fibres from pure cellulose and samples with added xylan showed almost identical curves. This was little surprising knowing that virtually all xylan remained dissolved in the spin bath and only the cellulose part of the carbohydrate solution formed filaments. However, fibres made from PG pulp give slightly higher residual mass.

Fig. 5c shows the yield upon carbonization in a tube furnace. In line with the TGA results, the CFs made from PG pulp (PG100) showed higher yields (∼13–14%) compared with CFs from dissolving pulp (E100) (∼8–10%). The difference in lignin content of the fibres was only minor (Table 5), indicating that the presence of hemicelluloses increased the char yield.

Carbonization without tensile stress on the fibres leads to fibres with low mechanical properties.^50^ Since the CFs were produced without applying any tension, the produced fibres were not strong enough for measuring the mechanical properties.

The carbon content of the obtained fibres was determined *via* elemental analysis and ranged from 82% to 86% at carbonization temperatures of 700–1100 °C (Table 7). This is lower than our previously reported values (88.6–91.7%) for cellulose and cellulose–lignin fibres.^9^ In the literature, different values are stated as minimum carbon content of CFs. A common convention however is 92 wt%.^3^ Our previously reported results were obtained using a different furnace, with a heating rate of 10 °C min^−1^. In this work we used heating rates of 2–5 °C min^−1^ (Table 2). The different heating programmes could influence the final carbon content of the CFs. Despite its absence in the starting materials (Table 3), a nitrogen content of 0.2–0.7% was detected in some CF samples. This could be explained by absorption phenomena occurring during the carbonization under nitrogen atmosphere.^5^

**Table 7:** Elemental analysis of CFs

	N [%]	±	C [%]	±	H [%]	±
E100 250 °C stab	0.0	0.0	41.6	0.5	6.0	0.0
E100 700 °C	0.2	0.0	85.8	0.2	1.4	0.0
E100 1100 °C	0.6	0.0	85.9	0.7	0.9	0.2
E100 1100 °C stab	0.7	0.0	82.1	0.4	0.3	0.0
PG100 250 °C stab	0.0	0.0	42.6	0.1	6.0	0.1
PG100 700 °C	0.2	0.0	83.4	0.7	1.4	0.0
PG100 1100 °C	0.5	0.0	84.7	0.5	0.4	0.1
PG100 1100 °C stab	0.5	0.0	84.3	0.8	0.3	0.0

**Fig. 6:**
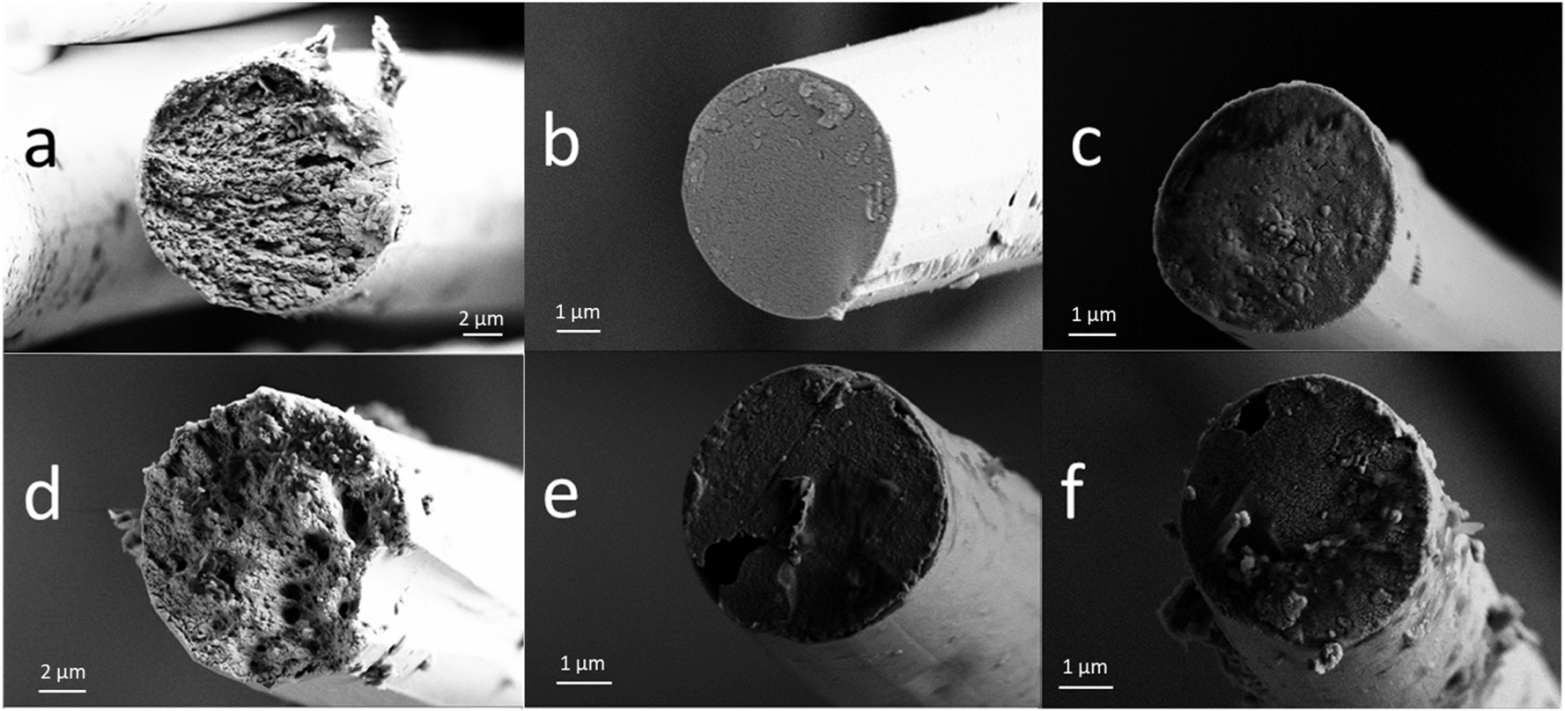
SEM images of CFs. (a) E100 DR6 250 °C, (b) E100 DR6 700 °C, (c) E100 DR6 1100 °C, (d) PG100 DR6 250 °C, (e) PG100 DR6 700 °C and (f) PG100 DR6 1100 °C.

In the SEM images (Fig. 6), the fibrillar structure is still visible after stabilisation at 250 °C (Fig. 6a and d). At 700 °C, there is a clear reduction in the diameter and the fibrillar structure has disappeared. Instead, the CFs show a dense, homogeneous structure and a smooth surface, with only minor defects sporadically distributed along the fibre (Fig. 6f).

**Fig. 7:**
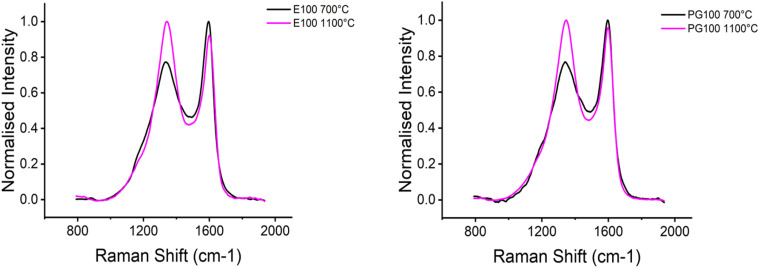
Raman spectra of selected CF samples.

Typically, Raman spectra of carbon fibres shows a G band at 1500–1630 cm^−1^ and a D band at ∼1355 cm^−1^. The D band is associated to aromatic rings in the carbon material. At lower carbonization temperatures, the carbon structures are still highly disordered, and the D band mainly corresponds to the probability of finding aromatic rings in the structure.^51^ For cellulosic precursors, the aromatisation starts at ∼700 °C.^52^ With increasing temperature, the carbon network gets more ordered and the D band intensity increases.^53^ The Raman results are depicted in Fig. 7. With increasing temperature, the D band intensity increased, indicating the presence of more ordered, graphite-like structures with increasing temperature. This agrees with previously published results.^54–56^

The crystalline structure was analysed *via* XRD and the results are summarised in Table 8. The interplane distance dc remains constant under the tested conditions and reaches a value of around 4 Å, which is close to the value reported for carbon fibers.^13,37,54^ The stabilization step used in the carbonization program (1100 °C samples) introduced a slight increase in the crystallite size and a slight decrease in the crystallinity index. However, the stabilized samples and the samples without the stabilization step show minor differences in the results. In agreement with results reported by Zahra *et al.*^54^ and Lundahl *et al.*,^37^ increasing the carbonization temperature caused the structure to expand laterally, with *L*_a_ increasing from 24 to 30 Å and from 28 to 32 Å, respectively, in parallel with the rise in crystallinity.

**Table 8:** Results from XRD analysis^a^^,^^b^^,^^c^

Sample	CRI [%]	*d*_c_ [Å]	*L*_*a*_ [Å]
E100 1100 Stab	16.1	3.99	30.3
E100 700	12.3	4.02	24.4
E100 1100	18.0	3.99	29.5
PG100 1100 Stab	16.8	4.00	33.0
PG100 700	11.8	4.01	28.2
PG100 1100	17.0	4.02	32.2

a CRI: Crystallinity index.

b *d*_c_: Interplane distance.

c *L*_*a*_: Crystallite size (in *a* direction).

## 4 Conclusions

Processes to isolate xylan from refined biomass can have a severe impact on the molecular weight of the hemicellulose chains. The refined xylan used in this study showed a significantly lower molar mass compared to hemicelluloses found in paper-grade pulp. This affects their solubility. In the case of dry-jet wet spinning of a cellulose–xylan mixture, only the cellulose coagulated in the aqueous spin bath whereas the xylan part remained dissolved. The dissolution and spinning of paper-grade pulp, however, resulted in mixed-polymer fibres with most of the original hemicelluloses being located in the fibre matrix. Upon carbonization in an oven, these fibres showed a slight increase in carbon yield. The resulting carbon fibres had almost identical properties as carbon fibres produced from dissolving-grade pulp. Thus, it is possible to resort to less expensive paper-grade pulp as feedstock to fully biobased carbon fibres. However, continuous carbonization preferably under constant tension would be needed to evaluate the full potential of these precursor filaments. The insights gained here align well with previous studies on the use of cellulose–lignin blends as feedstock for carbon fibres. This paves the way to our ultimate goal of spinning the entire wood matrix without refining into precursor fibres and convert them into wood-carbon fibres using a continuous carbonization line.

## Author contributions

Mikaela Trogen: conceptualization, investigation, formal analysis, visualization, writing – original draft. Petri Kilpeläinen: resources, writing – review & editing. Leena Pitkänen: investigation, formal analysis, writing – review & editing. Daisuke Sawada: investigation, formal analysis, writing – review & editing. Lukas Fliri: investigation, formal analysis, writing – review & editing. Inge Schlapp-Hackl: investigation, formal analysis, writing – review & editing. Michael Hummel: supervision, funding acquisition, conceptualization, resources, writing – review & editing.

## Conflicts of interest

There are no conflicts to declare.

## Supplementary Material

RA-015-D5RA07682J-s001

## Data Availability

The data that support the findings of this study are available from the corresponding author upon reasonable request. The raw datasets, including analytical results, have been stored securely and can be shared in compliance with institutional and journal guidelines. Supplementary information (SI): experimental details for carbohydrate and lignin analysis, molar mass distribution, mechanical properties, birefringence, and SEM. See DOI: https://doi.org/10.1039/d5ra07682j.
