# Supplementary material for: Cellulose–xylan composite fibres as precursors for carbon fibres
Source: RSC Adv. 2025 Dec 8;15(56):48061–71. doi: 10.1039/d5ra07682j (PMC12685134; doi:10.1039/d5ra07682j)
Supplement: RA-015-D5RA07682J-s001 [file RA-015-D5RA07682J-s001.pdf]

Supplementary Information (SI) for RSC Advances.
This journal is © The Royal Society of Chemistry 2025

Supplementary information

Cellulose-xylan composite fibres as precursors for carbon fibres

Mikaela Trogen[a], Petri Kilpeläinen[b], Leena Pitkänen[a], Daisuke Sawada[a], Lukas Fliri[a,c], Inge Schlapp-Hackl[a] and Michael Hummel[a]

a) Aalto University, School of Chemical Engineering, Department of Bioproducts and Biosystems, P.O. Box 16300, 00076 Aalto, Finland

b) Natural Resources Institute Finland (Luke), Latokartanonkaari 9, 00790 Helsinki, Finland

c) Department of Chemistry, Institute of Chemistry of Renewable Resources, BOKU University, Konrad-Lorenz-Strasse 24, 3430 Tulln, Austria

Contents

1 Experimental 2
1.1 Carbohydrate and lignin analysis 2
1.2 Molar mass distribution 2
1.3 Mechanical properties 2
1.4 Birefringence 2
1.5 SEM 3
2 References 4

1 Experimental

1.1 Carbohydrate and lignin analysis

The sample preparation for carbohydrate and lignin analyses were performed according to the NREL/TP-510-42618 standard and the samples were analysed using an ion chromatograph (ICS-3000 HPAEC-PAD) with a Dionex™ CarboPac™ PA20 column (ThermoFisher Scientific, USA). The eluent used was water and after each analysis, the column was washed with NaOH, followed by water for regeneration of the analysis conditions before the next injection. The acid-soluble lignin (ASL) was determined by using a Shimadzu UV 2550 spectrophotometer at 205 nm. The results for the pulp and the spun fibres were calculated according to procedures published by Janson[1].

1.2 Molar mass distribution

The molar mass distribution of carbohydrates was determined using a Dionex Ultimate 3000 HPLC module, with a Shodex differential refractive index (DRI) detector (model RI-101) and a Viscotek/Malvern SEC/MALS 20 multi-angle light-scattering (MALS) detector. The columns used were Agilent PLgel MIXED-A (x 4). As eluent, 0.9 % LiCl in DMAc was used with a flow rate of 0.75 mL/min. The samples were prepared using a solvent exchange procedure (water/acetone/DMAc) and finally dissolved in eluent (0.9 % LiCl/DMAc). The injection volume was 100 µL and sample concentration around 1 mg/mL. The detector constants (MALS and DRI) were determined using a narrow polystyrene sample (Mw = 96 000 g/mol, Đ = 1.04) dissolved in 0.9 % LiCl in DMAc.

A broad polystyrene sample (Mw = 248 000 g/mol, Đ = 1.73) was used for checking the detector calibration. The $\partial n/\partial c$ value of 0.136 mL/g was used for celluloses in 0.9 % LiCl in DMAc[2].

1.3 Mechanical properties

The mechanical properties of the PFs were measured using a Favigraph single-fibre tester (Textechno H. Stein GmbH & Co. KG, Germany) according to the standard SFS-EN 5079 with only minor modifications. The PFs were conditioned in 20 ± 2 ◦C and 65 ± 2 % RH overnight prior to the mechanical testing. The following conditions were used for the testing: Load cell 20 cN, gauge length 20 mm and test speed 20 mm/min. For each sample, 20 fibres were measured.

1.4 Birefringence

The birefringence was measured using a Zeiss Axio Scope A1 polarised light microscope with a Leica B 5λ-Berek tilting compensator. For the measurement, three filaments were taped on a microscope slide with two pieces of double-sided tape. The optical retardation was determined in duplicate from three selected spots on each filament. The linear density was measured using a TexTechno Favigraph single fibre tester. Also, for the linear density, three filaments were measured three times. The diameter was calculated assuming a density of 1.5 g/mL[3] for cellulose. Birefringence

Δn is defined as the retardation divided by the diameter. The total orientation was calculated by dividing Δn by 0.062, which is the maximum birefringence of cellulose[3].

1.5 SEM

A Zeiss Sigma VP instrument was used for the SEM imaging. To obtain an image of the cross section the fibres were prepared by cryofracture. A bundle of fibres was first taped across a shallow aluminium cup and the cup was filled with water. The cup was dipped into liquid nitrogen until the water froze, leaving the fibre bundles inside the ice cube. The ice cube was removed from the container and then cracked in the middle. The fractured fibre bundles were allowed to thaw and dry before attaching them onto the conductive support with carbon tape. The samples were sputter-coated with gold to ensure electric conductivity. The images were taken at 3 kV operating voltage.

2 References

1. J. Janson, *Paperi ja Puu*, 1970, **52**, 323–329.
2. A. Potthast, S. Radosta, B. Saake, S. Lebioda, T. Heinze, U. Henniges, A. Isogai, A. Koschella, P. Kosma, T. Rosenau, S. Schiehser, H. Sixta, M. Strlič, G. Strobin, W. Vorwerg and H. Wetzel, *Cellulose*, 2015, **22**, 1591–1613.
3. J. Lenz, J. Schurz and E. Wrentschur, *Holzforschung*, 1994, **48**, 72.
